# Virtual Interconsultations for Primary Care Referrals to Rheumatology: Real-World Implementation and Societal Cost-Avoidance Analysis in a Public Health System

**DOI:** 10.34172/ijhpm.9735

**Published:** 2026-08-18

**Authors:** Antonio Aznar, Marta Hernández-Díaz, Iván Ferraz-Amaro, Miguel Flores-Rodríguez, Jose María Viotti, María Vanesa Hernández-Hernández, Esmeralda Delgado-Frías, Alicia García-Dorta, Juan José Bethencourt Baute, Hiurma Sánchez-Pérez, Elisa Trujillo, María García-González, Enrique Gonzalez-Dávila, Federico Díaz-González

**Affiliations:** ^1^Servicio de Reumatología, Hospital Universitario de Canarias, La Laguna, Spain.; ^2^Departamento de Medicina Interna, Dermatología y Psiquiatría de la Universidad de La Laguna, La Laguna, Spain.; ^3^Departamento de Matemáticas, Estadística eInvestigación Operativa, IMAULL, Universidad de La Laguna, La Laguna, Spain.

**Keywords:** E-Consultation, Rheumatology, Primary Care, Cost-Avoidance, Referral Management, Spain

## Abstract

**Background:**

Rising demand for rheumatology care strains referral pathways between primary care and specialist services. We evaluated whether a virtual interconsultation (VIC) program, an asynchronous e-consult system integrated into the electronic health record (EHR), improved referral efficiency for new patients and generated net societal cost savings in a public health system.

**Methods:**

We conducted a retrospective study of 12 213 VICs (10 181 patients) submitted from primary care to a tertiary rheumatology service between January 2021 and June 2022. Extracted EHR data included demographics, referral reason, delay time (submission to specialist response), resolution time (minutes per case), and outcomes (virtual resolution vs. in-person referral). Referral efficiency was assessed by the proportion of referrals resolved without an in-person visit and time to specialist response. Descriptive and comparative analyses used Mann–Whitney U, chi-square, and student’s t tests. The economic evaluation used a cost-avoidance micro costing approach from a societal perspective. Robustness of the economic evaluation was assessed through one-way sensitivity analyses, probabilistic Monte Carlo simulations (>100 000 iterations), and threshold analysis (ie, the minimum virtual resolution rate at which the program achieves breakeven).

**Results:**

Patients were predominantly female (69%), mean age 54.8 years. Median specialist response time was 53.5 hours (interquartile range [IQR]: 20.6–122.3) and median resolution time 3.2 minutes (IQR: 1.6–8.6). Overall, 41.4% of referrals were resolved without an in-person visit. The program generated net societal savings of €206 280 over 18 months in the deterministic base case; the probabilistic analysis yielded a mean of €200 540 (95% credible interval: €142 289–€261 316) and a 100% probability of net savings. Absenteeism was the principal economic driver (highest Spearman correlation with net savings in Monte Carlo simulation, r=0.92). The breakeven virtual resolution rate was 5.6%.

**Conclusion:**

Integrating VICs into routine rheumatology referral pathways improved referral efficiency and produced substantial societal cost savings. These findings support scaling EHR-integrated VIC programs across publicly funded health systems to optimize specialist resources and enhance access to care.

## Background

**Key Messages**

**Implications for policy makers**

- Integrate virtual interconsultations (VICs) into electronic health records (EHRs) using standardized referral templates to ensure data quality, traceability, and timely specialist review.
- Allocate protected time within specialists’ work schedules for responding to VICs to support timely and consistent case management.
- Invest in training and change management for primary care physicians and specialists, emphasizing triage criteria, structured referrals, and effective digital communication.
- Strengthen governance and quality assurance through routine audits of referral appropriateness and regular reporting of VIC performance.

**Implications for the public**

Virtual interconsultations (VICs) enable primary care physicians to quickly obtain rheumatology advice, often within a few days, without requiring patients to travel to the hospital. This results in faster reassurance or treatment adjustments, fewer trips, and less time away from work or family obligations. By resolving many cases remotely, VICs ensure that in-person appointments are reserved for patients with more complex or urgent needs, thus improving fairness and prioritization of access to care. Since VICs are conducted securely within electronic health records (EHRs), patient information is protected while communication between healthcare professionals is enhanced. Overall, VICs provide a practical and convenient way to improve access to rheumatology care and optimize health system resource utilization.

Rheumatology services in many publicly funded health systems face rising demand and constrained specialist capacity. Traditional referral pathways often produce long waits, unnecessary in-person visits, and inefficient use of specialist time.^1,2^ Virtual interconsultations (VICs), an asynchronous e-consult system integrated into the electronic health record (EHR), have emerged as an innovative approach to improve referral efficiency. By providing rapid specialist input, diagnostic guidance, and therapeutic recommendations without requiring immediate in-person appointments, VICs reserve clinic slots for patients most likely to benefit.^3,4^ Beyond streamlining triage, VICs enhance care coordination, strengthen the rheumatology skills of primary care physicians, reduce unnecessary patient travel, and shorten waiting times by incorporating specialist input at the primary care level.^5-8^ Evidence from other medical specialties indicates that VICs reduce unnecessary specialist visits and shorten waiting times while preserving clinical safety and user satisfaction.^9-11^ Early studies in rheumatology suggest comparable benefits.^12,13^

 Although VICs have been shown to be an effective strategy,^3,13,14^ their implementation entails operational challenges. VICs may increase the administrative workload in primary care and asynchronous communication can introduce delays if initial data are incomplete or if successive clarifications are needed. These limitations are particularly relevant in complex or rapidly evolving cases.^7,15^ Despite evidence supporting the clinical utility of VICs,^3-13^ the economic impact of managing new rheumatology referrals from primary care through this model has not yet been systematically assessed from a cost-avoidance perspective.

 In 2013, the Rheumatology Department of the Hospital Universitario de Canarias (HUC), a tertiary center within the Canary Islands Health System, initiated a pilot VIC program in collaboration with primary care.^12^ The program was designed to optimize demand management and strengthen primary care physicians’ skills in managing rheumatic disorders.

 This study describes the implementation of an EHR-integrated VIC program at a tertiary center and evaluates its effectiveness and societal cost-avoidance for new primary-care rheumatology referrals.

## Methods

###  Study Design and Setting

 We conducted a retrospective observational study of all new primary care referrals to rheumatology managed through the VIC pathway at the University Hospital of the Canary Islands (HUC), Spain, between January 4, 2021, and June 30, 2022.

 HUC is a tertiary hospital serving approximately 463 751 inhabitants across northern Tenerife Island and La Palma island. The Rheumatology Department comprised one head of department, 14 attending physicians, and one resident per year.^16^ Weekly clinical activity at the Rheumatology Department was structured into 35 outpatient clinic modules: 20 located at the hospital and 15 distributed across three specialized care centers (SCCs) within the health area. In 2022, 22 183 outpatients were seen in-person (60.4% hospital, 39.6% SCCs), 7500 via VIC, 2133 in the day hospital, and 42 admitted as inpatients^16^ (Supplementary file 1, Table S1).

###  Virtual Interconsultation Workflow and Organization

 Primary care physicians submitted VICs via the EHR platform integrated within the SAP system (Systems, Applications, and Products in Data Processing, Walldorf, Germany). At SCCs, rheumatology consultations were organized in structured morning sessions: 08:30-09:30, up to 10 VICs; 09:30-11:30, eight new in-person patients; and 11:30-13:30, up to 12 follows-up or telephone visits. Each rheumatologist managed VICs originating from primary care centers (PCCs) within their SCC catchment area. When an in-person evaluation was required, the same specialist who reviewed the VIC scheduled the appointment directly to ensure full traceability. When an in-person visit was deemed unnecessary, the primary care physician received a VIC report that included the presumptive diagnosis, recommended additional tests, and management suggestions.

###  Data Sources and Variables

 The study database was obtained from the SAP system used for overall management at HUC. SAP Sanidad integrates clinical, administrative, and financial modules and functions as a secure EHR platform with direct access to clinical information, laboratory results, and imaging studies.

 Data extracted from mandatory administrative records in SAP by the hospital’s information technology department using structured queries included patient demographics, reason for referral (as assessed by rheumatologists in predefined diagnostic categories, see below), delay time (hours from submission to specialist response), resolution time (minutes from opening to closure of the VIC; see Figure S1 of Supplementary file 1), outcome (resolved virtually vs. referred to in-person consultation), and number of VICs per patient.

 Between November 1, 2021, and June 30, 2022, rheumatologists classified the reasons for 5381 VICs (94.3% of the 5703 evaluated during this period) into the following categories: soft tissue disorders (shoulder tendinitis, trochanteritis, epicondylitis, and plantar fasciitis); mechanical arthralgia (mechanical pain without radiological evidence of osteoarthritis, excluding low back and neck pain); lower back and neck pain; peripheral osteoarthritis with radiological evidence; joint inflammation; suspected connective tissue disease (cutaneous lesions, aphthae, or Raynaud’s phenomenon); osteoporosis (pathological bone mineral density or low-energy fracture); fibromyalgia; administrative consultations; treatment-, laboratory tests-, or imaging studies-related queries.

###  Outcome Definitions

 The following outcomes were assessed. Referral efficiency was evaluated through (*a*) virtual resolution rate, defined as the proportion of referrals managed without an in-person visit; and (*b*) delay time, defined as the number of hours from VIC submission by the primary care physician to the specialist response. The economic impact was measured as net societal savings, defined as the economic savings from avoided in-person visits, reduced patient travel, and reduced work absenteeism, minus the direct cost of specialist time for VIC review. Resolution time, defined as the elapsed time in minutes from the moment the rheumatologist opened the VIC to the moment the response was finalized, and the case was closed (See Figure S1), was used to estimate specialist time costs.

###  Economic Evaluation (Cost-Avoidance Analysis)

 The economic analysis adopted a societal perspective over the 18-month study horizon (currency year: 2022 €), following the Consolidated Health Economic Evaluation Reporting Standards 2022 recommendations.^17^ We used a cost-avoidance microcosting approach, comparing the resource use of virtual consultations with that of conventional in-person visits.

 Costs were estimated for three components. Direct healthcare costs included specialist time, valued at the average employer cost per hour of a healthcare specialist within the Canary Islands Health System.^18^ Direct non-healthcare costs included patient transportation, estimated as the mean round-trip public-transport (bus) fare from the patient’s health area to the hospital.^19^ Indirect costs (productivity losses due to work absenteeism) were estimated using the human capital approach, calculated as the product of: the number of working-age patients (25–65 years) whose referral was resolved virtually, the regional employment rate (81%),^20^ the estimated time saved per avoided in-person visit (2.5 hours), and the average employer cost per effective working hour (€19.55).^21^ The 2.5-hour estimate comprised average travel time, 30-minute waiting-room time, and 30-minute consultation duration. Details of the economic methods and their limitations are provided in the Supplementary Text (See Supplementary file 1).

 To test the robustness of the findings, deterministic one-way sensitivity analyses, in which each parameter is varied individually across a plausible range while all others are held at base-case values, were performed on key parameters. Probabilistic Monte Carlo simulations (>100 000 iterations) were conducted using the following input distributions: patient time (triangular, 1.0-4.0 hours), specialist VIC time (triangular, 1.8-7.2 minutes), and employment rate (beta distribution, mean 0.81, standard deviation ≈ 0.05) (See Table S2). Triangular distributions were selected for time-based parameters because, unlike uniform distributions (which assign equal probability to all values in a range), they allow specification of a minimum, a maximum, and a most likely (mode) value, better reflecting operational parameters where extreme values are less frequent than central ones. This is a standard choice in health economic modeling when empirical data are limited to summary statistics rather than full distributional data. These simulations generated the probability of achieving net savings, 95% credible intervals, and correlation coefficients identifying the variables with the greatest influence on savings. Breakeven (threshold) analysis was also performed to identify the minimum virtual resolution rate at which the VIC program’s savings equal its costs (ie, net savings = €0). All monetary values are reported in euros (€).

###  Statistical Analysis

 A descriptive analysis was performed for demographic and VIC-related variables. Continuous variables were expressed as mean ± standard deviation when normally distributed, or as median with interquartile range (IQR) when non-normally distributed. Categorical variables were presented as absolute frequencies and percentages. Comparisons of means between two groups were performed using the independent-samples student’s *t* test, while the Mann–Whitney U tests were applied for medians. Proportions were compared using Pearson’s chi-square test or Fisher’s exact test. Associations between tertiles of resolution time and the need for an in-person appointment were assessed using chi-square. Delay and resolution times between patients with and without appointments were compared using the Mann–Whitney U test. To assess the independent effect of potential confounders on VIC outcomes, a multivariable logistic regression model was fitted with in-person appointment (yes/no) as the dependent variable. The model included resolution time tertile, rheumatologist, and referral reason as main-effect covariates. Global significance for each variable was assessed using the Wald chi-square statistic. Results are reported as odds ratios (ORs) with 95% confidence intervals.

 All statistical analyses were performed using SPSS (version 29 IBM SPSS, Armonk, NY). Probabilistic sensitivity analyses and Monte Carlo simulations of the program’s net economic savings were performed using R (version 4.2.0; R Foundation for Statistical Computing, Vienna, Austria). Random variates were drawn from triangular and beta distributions; Spearman rank correlation coefficients were computed to identify the most influential parameters. Results were considered significant if *P*< .05. Graphs were generated using GraphPad Prism 10 version 10.4.1, GraphPad Software, San Diego, California, USA (https://www.graphpad.com/).

## Results

###  Activity and Sample Characteristics

 During the 18-month study period, the Rheumatology Department received 13 886 VICs; 12 213 (87.9%) originated from PCCs were included in this analysis, corresponding to 10 181 unique patients. Patients were predominantly female (7029; 69.0%) with mean age 54.8 ± 15.4 years and median 55 years (IQR 45-65). Age distribution: <25 years 3.9%; 25-45 years 19.4%; 45-65 years 50.5%; 65-75 years 15.8%; ≥75 years 10.5%. No significant sex-related differences in age distribution were observed (Figure S2).

###  Temporal Pattern, Reasons for Virtual Interconsultations and Repeat Consultations

 The mean number of VICs received from PCCs was 32.7 ± 8.1 per day. Lower volumes were observed in January, July, and December (Table S3). There was no significant difference in the average number of VICs across weekdays (*P* = .999). Figure S3 shows the reasons for VICs, as classified by the rheumatologist (See Methods section). The most frequent referral categories were soft tissue disorders (22.8%), mechanical arthralgia (21.7%), lower back and neck pain (15.1%), peripheral osteoarthritis (11.3%), and joint inflammation (11.2%). Less frequent categories (each <5%) included suspected connective tissue disease (4.6%), suspected osteoporosis (3.7%), fibromyalgia (3.0%), and administrative, treatment, laboratory, and imaging queries (6.6%). Most patients (8502; 83.5%) had one VIC; 1379 (13.5%) had two VICs; and 300 (2.9%) had three or more (Figure S4).

###  Timeliness and Resolution Metrics

 Median delay time from VIC submission to specialist response was 53.5 hours (IQR: 20.6–122.3). No meaningful correlation was observed between delay and resolution times (r = −0.003, *P* = .709).

 Median resolution time per VIC was 3.2 minutes (IQR: 1.6-8.6); 80% of VICs were resolved in less than 12.2 minutes (Table 1). Resolution time was distributed across tertiles as follows: ≤1.6 minutes (23.8%), 1.6-8.6 minutes (51.2%), and ≥8.6 minutes (24.9%).

**Table 1:** Percentile Distribution of Resolution Time Overall and Stratified by In-Person Appointment Outcome

**Percentile**	**Total (min)**	**With In-Person Appointment (min)**	**Without In-Person Appointment (min)**
10	1.2	1.5	1.2
20	1.6	1.6	1.2
30	1.8	1.8	1.5
40	2.3	2.2	2.7
50	3.2	2.7	4.0
60	4.6	3.8	5.7
70	6.7	5.5	7.8
80	12.2	10.6	14.0
90	293.3	219.9	411.8

Abbreviation: VIC, virtual interconsultation. Resolution time: elapsed time in minutes from the moment the rheumatologist opened the VIC to the moment the response was finalized and the case was closed. Values represent the resolution time (in minutes) at each percentile of the distribution. The 50th percentile corresponds to the median. With in-person appointment: VICs that resulted in scheduling a face-to-face consultation. Without in-person appointment: VICs resolved virtually without requiring an in-person visit.

 Resolution times varied among clinicians: two rheumatologists resolved more than 42% of their VICs in ≤1.6 minutes, whereas two others required ≥8.6 minutes for at least 35% of their cases (Table 2; Supplementary file 1, Table S4).

**Table 2:** Distribution of Resolution Time Overall and by Tertiles, Stratified by In-Person Appointment Outcome

	**Median (IQR), min**	***P***	**Resolution Time Tertiles, n (%)**			***P***
			**≤1.6 min**	**1.6–8.6 min**	**≥8.6 min**	
**Total**	3.2 (1.6; 8.6)		2912 (23.8)	6258 (51.2)	3045 (24.9)	<.001
Without appointment	2.7 (1.7; 7.1)	<.001	1783 (61.2)	2380 (38.0)	1589 (52.2)	
With appointment	4.0 (1.3; 9.7)		1129 (38.8)	3878 (62.0)	1456 (47.8)	

Abbreviation: IQR, interquartile range. Resolution time tertiles: ≤1.6, 1.6–8.6, and ≥8.6 minutes. *P* values: Mann–Whitney U test comparing medians between groups, and chi-square test for the association between resolution time tertiles and appointment outcome.

###  Outcomes and Associated Factors

 Associations between VIC outcomes (in-person appointment vs. no appointment) and key variables, including delay time, resolution time, referral reason, and number of VICs per patient, were analyzed. Overall, 4211 of 10 181 patients (41.4%) were managed without an in-person visit. Patients scheduled for in-person appointments had a slightly shorter median delay time than those not referred (51.7 hours, IQR 20.7-117.6 vs. 57.2 hours, IQR 20.6-137.7; *P* = .008). VICs resulting in appointments also had shorter median resolution times than those resolved virtually (2.7 minutes, IQR 1.7-7.1 vs. 4.0 minutes, IQR 1.3-9.7; *P* < .001). Among VICs resolved without an appointment, 61.2% were completed in ≤1.6 minutes, whereas 62.0% of those resulting in an appointment required 1.6-8.6 minutes for resolution (*P* < .001; Table 2). Resolution times and outcomes by referral reason are summarized in Table 3. Inflammatory presentations (suspected arthritis, connective tissue disease) had referral rates exceeding 67%, whereas degenerative or nonspecific conditions (osteoarthritis, fibromyalgia, mechanical pain) had referral rates below 40%. Referral rates increased progressively with the number of VICs per patient (Figure S4). In multivariable logistic regression, resolution time tertile, referral reason, and rheumatologist were all independently associated with the likelihood of an in-person appointment (*P* < .001 for all; Table S5).

**Table 3:** Time Invested and Number of Appointments According to the Reason for the VIC

**Reason for VIC**	**% Of Total Patients**	**Resolution Time Percentile (min)**			**% Of Total Resolution Time**	**% Of Consultations Requested by Reason**			**% Of Appointments by Reason**
		**25**	**50**	**75**		**1 (n = 8502)**	**2 (n = 2758)**	**≥3 (n = 955)**	
Soft tissue	22.8	1.9	3.4	10.0	17.3	55.4	27.5	17.1	58.9
Mechanical arthralgias	21.7	2.0	3.4	8.1	16.4	57.7	29.7	12.6	58.3
Lower back and neck pain	15.1	2.2	5.8	10.2	19.5	62.5	27.0	10.5	37.1
Peripheral osteoarthritis	11.3	1.8	5.8	9.8	14.6	55.1	31.2	13.7	34.7
Arthritis	11.2	2.4	4.7	11.6	11.7	47.2	36.1	16.7	70.5
Suspected connective tissue disorder	4.6	2.4	4.6	9.5	4.7	64.0	29.3	6.7	67.3
Osteoporosis	3.7	2.3	5.8	10.7	4.8	58.4	32.0	9.6	45.6
Fibromyalgia	3.0	3.7	6.2	14.7	4.1	49.1	28.3	20.6	38.4
Other^a^	6.6	2.2	4.7	9.6	6.9	56.5	29.8	13.7	29.2

Abbreviation: VIC, virtual interconsultation. ^a^Consultations regarding imaging, laboratory tests, treatments, dislocations, post-traumatic conditions, or administrative issues.

###  Economic Results

 Implementation of the VIC program generated a total cost of €25 303.33 and yielded net savings of €206 280.44. The main contributors to savings were reductions in employer absenteeism (€118 271.24), specialist time allocated to face-to-face visits (€79 106.37), and patient transport costs (€34 208.16). On average, the saving per VIC response was €16.89 (Table 4). Sensitivity analyses confirmed the robustness of these findings across variations in key parameters, including resolution rate (base case: 42%), average absenteeism (2.5 hours), employment rate (81%), specialist hourly cost, and the exclusion of private transport or indirect costs. In one-way sensitivity analysis, absenteeism exerted the greatest influence on net savings (r = 0.92), followed by specialist resolution time (r = 0.26), whereas unemployment rate was negatively correlated (r = −0.27) (Figure A). Probabilistic Monte Carlo simulations (>100 000 iterations) showed a 100% probability of net savings. The distribution of simulated outcomes was tightly centered around the observed mean (€200 540.5) and median (€200,010.8), with a 95% credible interval of €142 289.1 to €261 316.1. Threshold analysis indicated a breakeven resolution rate of 5.6%, far below the observed rate of 41.6% (Figure B).

**Table 4:** Input Parameters, Formulas, and Economic Results of the Virtual Interconsultation Program

**Variable descriptions**	**Value of Variables**	**Formulas**	**Economic and Time-Related Results**
A. Number of patients^a^	10 181		
B. Number of patients of working age (25-65 years)	7116		
C. Number of VIC^a^	12 213		
D. Average time of in-person consultation (h)	0.5		
E. Average time per patient per in-person consultation (h)^b^	2.5		
F. Average time per physician per VIC (h)^a^	0.056		
G. Hourly cost of worker (€)^c^	19.55		
H. Hourly cost of specialist (€)^d^	37		
I. Consultations that can be resolved by VIC (%/100)^a^	0.42		
J. Average cost of public transport (€)^e^	8		
K. Average employment rate in the Canary Islands in 2021 (%/100)^f^	0.81		
1. Medical time costs of VICs (€)		C×F×H	25 305.33
2. Savings to employers from reduced absenteeism (€)		B×K×I×E×G	118 271.24
3. Patient transportation cost savings (€)		A×J×I	34 208.16
4. Economic savings on medical time (€)		A×I×D×H	79 106.37
5. Final result (€)		(2+3+4)-1	206 280.44
6. Per-VIC savings (€)		5/1	16.89
7. Total cost of assistance in the absence of the VIC program		(B×K×E×G) + (A×J) + (A×D×H)	551 510.04

Abbreviation: VIC, virtual interconsultation. ^a^ Values obtained in this study. ^b^ Includes average transport time,^19^ waiting time in the consultation room (30 min), and consultation time for a new patient (30 min). ^c^ Average employer cost of a worker in the Canary Islands, including salary, contributions, and other costs.^21^ ^d^ Average employer cost of a medical specialist in the Canary Islands, including salary, contributions, and other costs.^18^ ^e^ Round-trip bus fare from Puerto de la Cruz (midpoint of the health area) to the hospital.^19^ ^f^ Based on the average unemployment rate in the Canary Islands in 2021, estimated at 19% of the population aged 25–65 years.^20^

**Figure:**
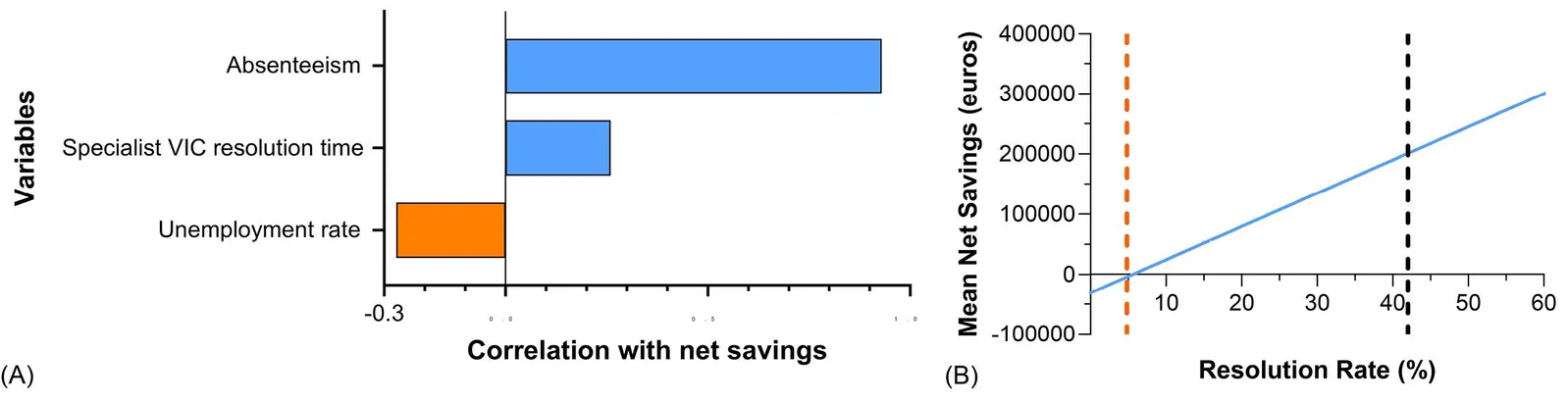
(A) Tornado Plot Showing the Effect of Key Parameters on Net Savings. Bars represent the range of net savings when each parameter is varied and are ordered by impact achieve breakeven (5.6%). (B) Relationship Between Resolution Rate (%) and Mean Net Savings (€). The orange dashed vertical line indicates the minimum resolution rate required for the VIC program to achieve breakeven (5.6%); the black dashed vertical line marks the observed resolution rate (41.4%). Abbreviation: VIC, virtual interconsultation.

## Discussion

 This large, real-world evaluation demonstrates that an asynchronous, EHR-integrated VIC program can resolve a substantial proportion (41.4%) of new primary care rheumatology referrals without requiring an in-person visit, while maintaining rapid specialist response times and generating meaningful societal cost avoidance. The economic advantage remained robust across deterministic and probabilistic sensitivity analyses and was primarily driven by reductions in productivity losses. This underscores the broader societal value of digital triage models in publicly funded health systems.

 The demographic and diagnostic patterns observed in this study align with the known epidemiology of rheumatic and musculoskeletal disorders, which predominantly affect middle-aged women.^22-24^ Seasonal fluctuations in VIC volume likely reflect organizational factors rather than changes in disease burden, emphasizing the need for consistent specialist coverage throughout the year. The diagnostic spectrum mirrored that of typical primary care referrals to rheumatology,^25-27^ with soft tissue disorders, mechanical arthralgia, and spinal pain accounted for most VICs and required relatively short review times. In contrast, suspected inflammatory arthritis, connective tissue disease, and fibromyalgia, which require the exclusion of alternative diagnoses, required longer assessments.^28,29^ This supports the role of VICs as an effective triage mechanism that retains straightforward degenerative or mechanical conditions in primary care while prioritizing inflammatory cases for in-person evaluation. However, our pattern of referrals differs from those reported in other virtual programs: in the Canadian BASE e-Consult service, osteoporosis was the most frequent reason (22%) followed by polyarthralgias and polyarthritis^13^; in a US safety-net setting, inflammatory arthritis dominated the referral mix^30^; and in a Spanish tertiary hospital, administrative and treatment-management queries accounted for 36% of e-consultations.^31^ These differences likely reflect variation in referral criteria, population characteristics, and program design.

 The operational performance was favorable. The median response time was just over two days, and most VICs were resolved in under 12 minutes. These results align with previous teleconsultation studies^13,30^ and suggest that once information is organized within the EHR, specialists can make rapid and focused decisions. VICs that resulted in in-person visits had shorter resolution times, likely due to clearer referral criteria. In contrast, VICs that were resolved virtually often required more detailed diagnostic assessments and/or management guidance, which naturally extend the time required for response. The observation that referral rates increased among patients with repeated VICs suggests that recurrent electronic queries may serve as an implicit signal of persistent or evolving disease.

 Integrating VICs into the existing EHR platform improved transparency, traceability, and workflow integration. However, variability in resolution times among clinicians indicates opportunities for standardization. Multivariable logistic regression confirmed that rheumatologist, together with resolution time and referral reason, was independently associated with the likelihood of an in-person appointment. International guidance emphasizes the importance of defined competencies in tele-rheumatology,^32,33^ continuous professional development focused on digital communication and triage may enhance the consistency and reliability of VIC programs. Inter-clinician variability could also influence per-VIC costs; although a full clinician-level cost analysis was beyond the scope of this study, the sensitivity analyses partially captured this variability by including specialist resolution time as a key parameter in the Monte Carlo simulation.

 The economic findings have important implications for health service planners. Although the VIC program required modest direct investment, it produced substantial savings through avoided in-person visits, reduced travel, and lower absenteeism. The breakeven virtual resolution rate was only 5.6%, far below the observed rate of 41.4%, indicating strong financial resilience. The significant impact of productivity losses on overall savings underscores the importance of adopting a societal perspective when evaluating outpatient care models, especially for working-age populations. The Monte Carlo simulation, which varied key parameters simultaneously across plausible ranges, consistently yielded a 100% probability of net savings, supporting the overall robustness of the economic conclusions.

 Several policy considerations emerge from these findings. Integrating VICs within EHR systems ensures data quality, traceability, and efficient specialist review. Allocating protected specialist time is essential to maintaining timely responses and avoiding workflow bottlenecks.^34-37^ Structured training and change management initiatives focused on triage criteria, standardized referral templates, and effective digital communication are critical for both primary care and specialist clinicians to sustain program performance. Routine audits of referral appropriateness and transparent reporting can strengthen governance, support continuous quality improvement, and enhance accountability.^38-41^

 This study has limitations. Its single-center design may limit its applicability to health systems with different organizational structures, geographic characteristics, or digital infrastructures; multicenter validation studies should be a priority for future research. Additionally, patient and clinician satisfaction were not assessed, which limits insights into acceptability and perceived quality. Future studies should assess patient and clinician satisfaction and explore barriers and facilitators of VIC implementation. Triage appropriateness was not formally reaudited, raising the possibility of misclassification; future programs should incorporate structured clinical independent audits as part of quality assurance. The economic analysis relied on simplifying assumptions regarding working-age definitions, employment patterns, and transportation modes, which may have led to the underestimation or overestimation of costs in specific subgroups. Finally, direct program costs were calculated based solely on the time rheumatologists spent responding to VICs. Inter-case intervals were not included, which could have led to an underestimation of personnel costs. System-level overhead costs were not included, as the SAP system was already in place for broader hospital management. The cost-avoidance framework does not include a comparator arm with randomized allocation; estimated savings represent costs avoided rather than measured differences from a controlled comparison. Nevertheless, the large sample size, real-world setting, integrated electronic platform, and robust analytic approach provide strong evidence that VICs can improve referral pathways and generate meaningful societal benefits.

## Conclusions

 Overall, these findings support integrating and scaling VIC programs within publicly funded health systems as a strategy to improve access, optimize specialist resources, and reduce societal costs.

## Acknowledgements

 We thank the primary care physicians and administrative staff who supported the implementation of the VIC program, as well as the SAP data management team for facilitating data extraction. We are especially grateful to Dr. Sagrario Bustabad Reyes for her pivotal role in the early implementation of the VIC program and for her valuable critical reading of this work.

## Disclosure of artificial intelligence (AI) use

 AI-assisted tools such as Claude (Anthropic), Microsoft Copilot (Microsoft), and DeepL (DeepL SE) were used solely for language editing, grammar and style improvement and formatting of references. The authors reviewed and edited all AI-assisted output, verified that every reference is accurate and corresponds to its original source, checked the text for plagiarism, and take full responsibility for the integrity and content of the manuscript.

## Ethical issues

 The study was conducted in accordance with the principles of the Declaration of Helsinki and with applicable Spanish and European data-protection legislation (Regulation (EU) 2016/679 [GDPR]; Organic Law 3/2018 [LOPDGDD]; Law 14/2007 on Biomedical Research). The study protocol was reviewed and approved by the Research Ethics Committee (CEIm) of the Complejo Hospitalario Universitario de Canarias (CHUC) (reference CHUC_2025_143). Given the retrospective design and the use of pseudonymized data, the Committee waived the requirement for informed consent.

## Conflicts of interest

 Authors declare that they have no conflicts of interest.

## Disclaimer

 The views expressed are solely those of the authors and do not necessarily reflect the policies or positions of the Hospital Universitario de Canarias (HUC), the Universidad de La Laguna, or the Servicio Canario de la Salud (Canary Islands Health Service).

## Supplementary files


Supplementary file 1 contains Tables S1-S5, Figures S1-S4, and Supplementary Text.
