# Supplementary material for: Virtual Interconsultations for Primary Care Referrals to Rheumatology: Real-World Implementation and Societal Cost-Avoidance Analysis in a Public Health System
Source: Int J Health Policy Manag. 2026 Aug 18;15:9735. doi: 10.34172/ijhpm.9735 (PMC13770224; doi:10.34172/ijhpm.9735)
Supplement: Supplementary file 1 [file ijhpm-15-9735-s001.pdf]

Article title: Virtual Interconsultations for Primary Care Referrals to Rheumatology: Real-World Implementation and Societal Cost-Avoidance Analysis in a Public Health System

Journal name: International Journal of Health Policy and Management (IJHPM)

Authors' information: Antonio Aznar[1], Marta Hernández-Díaz[1], Iván Ferraz-Amaro[1,2], Miguel Flores-Rodríguez[1], Jose María Viotti[1], María Vanesa Hernández-Hernández[1], Esmeralda Delgado-Frías[1], Alicia García-Dorta[1], Juan José Bethencourt Baute[1], Hiurma Sánchez-Pérez[1], Elisa Trujillo[1,2], María García-González[1], Enrique Gonzalez-Dávila[3], Federico Díaz-González[1,2]*

[1]Servicio de Reumatología, Hospital Universitario de Canarias, La Laguna, Spain.

[2]Departamento de Medicina Interna, Dermatología y Psiquiatría de la Universidad de La Laguna, La Laguna, Spain.

[3]Departamento de Matemáticas, Estadística eInvestigación Operativa, IMAULL, Universidad de La Laguna, La Laguna, Spain.

***Correspondence to:** Federico Díaz-González; Email: jfdiazg@ull.edu.es

Citation: Aznar A, Hernández-Díaz M, Ferraz-Amaro I, et al. Virtual interconsultations for primary care referrals to rheumatology: real-world implementation and societal cost-avoidance analysis in a public health system. Int J Health Policy Manag. 2026;15:9735. doi:10.34172/ijhpm.9735

Supplementary file 1

Supplementary Table 1. Catchment Area, Human Resources, and Clinical Activity of the Rheumatology Service *

**Care area**		465,751 inhabitants
**Human Resources**	Head of Service	1
	Area Specialist Physicians	14
	Resident/year	1
**Care (**labor shifts/week**)**	Outpatient Consultations – Hospital	20
	Outpatient Consultations – Area	15
	Biological Therapy	5
	Techniques	4
	Ward/Emergency Room	5
**Care activity** (patients/year)	Hospital Consultations	13,401
	SCC Consultations	8,782
	Virtual Consultations	7,500
	Day Hospital Consultations	2,133
	Inpatient Care	42 cases in Rheumatology<br>75 inpatient interconsultations

*https://www3.gobiernodecanarias.org/sanidad/scs/scs/as/tfe/28/memorias/2022/index.html

Supplementary Table 2. Input Parameters for the Economic Model and Probabilistic Sensitivity Analysis

Parameter	Base case	Distribution	Min	Mode	Max	Source/Rationale
Parameters varied in Monte Carlo simulation						
Patient time per visit (h)	2.5	Triangular	1.0	2.5	4.0	Transport + waiting + consultation time
Specialist VIC time (min)	3.4	Triangular	1.8	3.4	7.2	Observed median; min/max from P10–P90
Employment rate	0.81	Beta (α≈49.1, β≈11.5)	—	0.81	—	INE 2021 Canary Islands; SD ≈ 0.05
Fixed parameters (varied in one-way deterministic sensitivity analysis)						
Specialist hourly cost (€)	37	Fixed	—	37	—	CHS salary tables
Worker hourly cost (€)	19.55	Fixed	—	19.55	—	INE ETCL
Transport cost (€)	8	Fixed	—	8	—	Public bus round-trip
Virtual resolution rate	0.42	Fixed	—	0.42	—	Observed; varied in threshold analysis

Triangular distributions for time-based parameters, defined by min, mode, and max values, and beta distribution for the employment rate, bounded between 0 and 1 as is conventional for proportions, were used for the Monte Carlo simulation probabilistic sensitivity analysis (>100,000 iterations). Fixed parameters were explored in one-way deterministic sensitivity analyses.

INE = Instituto Nacional de Estadística; CHS = Canary Islands Health System; ETCL = Encuesta Trimestral de Coste Laboral; P10–P90 = 10th–90th percentile range from observed data.

Beta distribution parameterized from mean and approximate SD using the method of moments (α ≈ 49.1, β ≈ 11.5 for mean = 0.81, SD ≈ 0.05).

Supplementary Table 3. Monthly counts and daily means of VICs referred from primary care during the study period

Year	Month	Nº of VIC	Mean by day	SD
2021	January	587	30.9	5.4
	February	626	32.9	7.9
	March	806	35.0	5.3
	April	774	38.7	6.0
	May	751	35.8	7.4
	June	806	36.6	6.4
	July	678	30.8	6.8
	August	670	31.9	6.2
	September	732	33.3	5.3
	October	693	34.6	6.5
	November	690	32.9	7.7
	December	470	24.7	8.6
2022	January	389	19.4	6.4
	February	642	33.8	7.6
	March	809	35.2	8.8
	April	681	35.8	5.7
	May	704	33.5	9.8
	June	705	32.0	6.0
**Total**		12.213	32.7	8.1

SD: standard deviation; VIC: virtual interconsultation

Supplementary Table 4. Distribution of resolution time tertiles by rheumatologist

Rheumatologist	≤1.6 min (%)	1.6–8.6 min (%)	≥8.6 min (%)
M1	16.9	61.0	22.1
M2	12.2	57.3	30.5
M3	6.1	58.0	35.9
M4	13.8	69.5	16.7
M5	10.9	68.6	20.5
M6	42.1	34.6	23.3
M7	14.9	65.1	20.0
M8	32.7	53.8	13.5
M9	63.4	33.6	3.0
M10	28.4	40.5	31.0
M11	13.3	58.4	28.2
M12	16.4	41.9	41.7

Values represent the percentage of each rheumatologist's VICs falling within each resolution time tertile (≤1.6, 1.6–8.6, and ≥8.6 minutes). Rheumatologists are anonymized (M1–M12).

Pearson's chi-square test for the association between rheumatologist and resolution time tertile: $p < 0.001$ (df = 22).

Supplementary Table 5. Multivariable logistic regression model for in-person appointment outcome

Variable	*B*	SE	Wald χ²	*df*	*p*	OR	95% CI
**Resolution time (ref. ≤1.6 min)**			197.783	2	<0.001		
(1.6; 8.6]	1.571	0.122	167.195	1	<0.001	4.812	3.792–6.106
>8.6	0.865	0.132	42.767	1	<0.001	2.376	1.833–3.079
**Rheumatologist**			72.314	12	<0.001		
**Referral reason**			223.790	8	<0.001		
Constant	−0.732	0.138	27.992	1	<0.001	0.481	

Dependent variable: in-person appointment (1 = yes, 0 = no). Main-effects model without interactions. Rheumatologist (12 df) and referral reason (8 df) are shown as global Wald tests.

OR = odds ratio; CI = confidence interval; SE = standard error; df = degrees of freedom; B = regression coefficient.

.

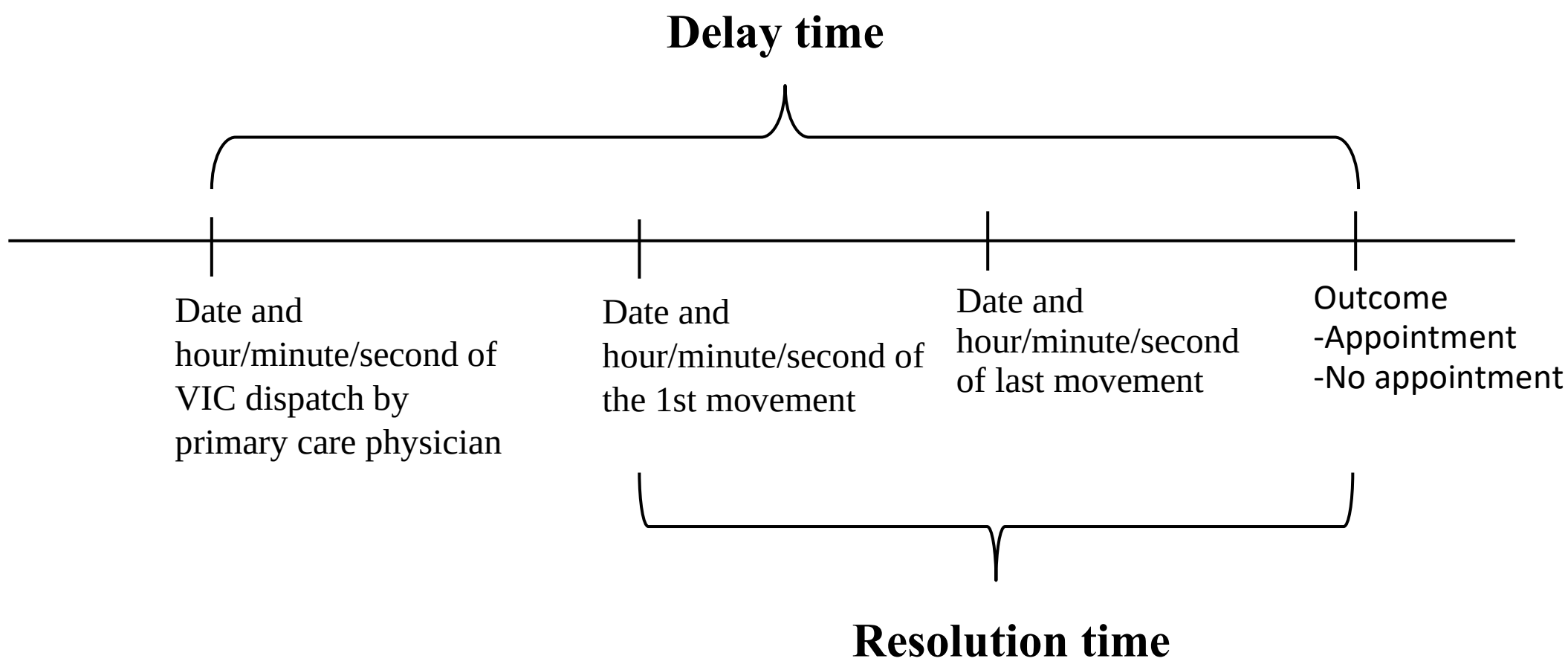

Supplementary Figure 1. Information captured in the electronic system and definitions of time variables

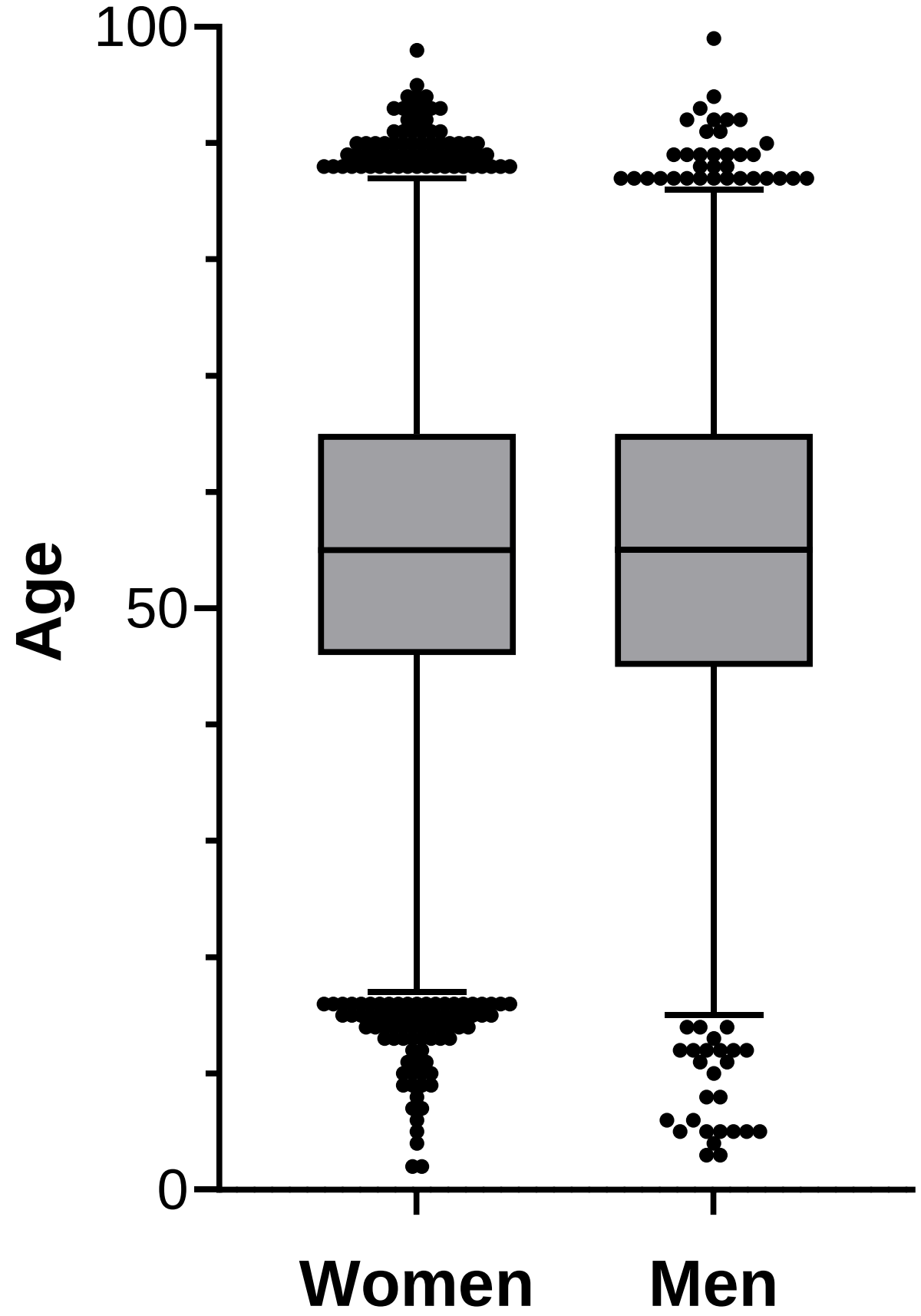

Supplementary Figure 2. Age distribution by sex. Box-and-whisker plots show median, interquartile range (25th–75th percentiles), whiskers extending to the 1st–99th percentiles, and individual dots representing outliers.

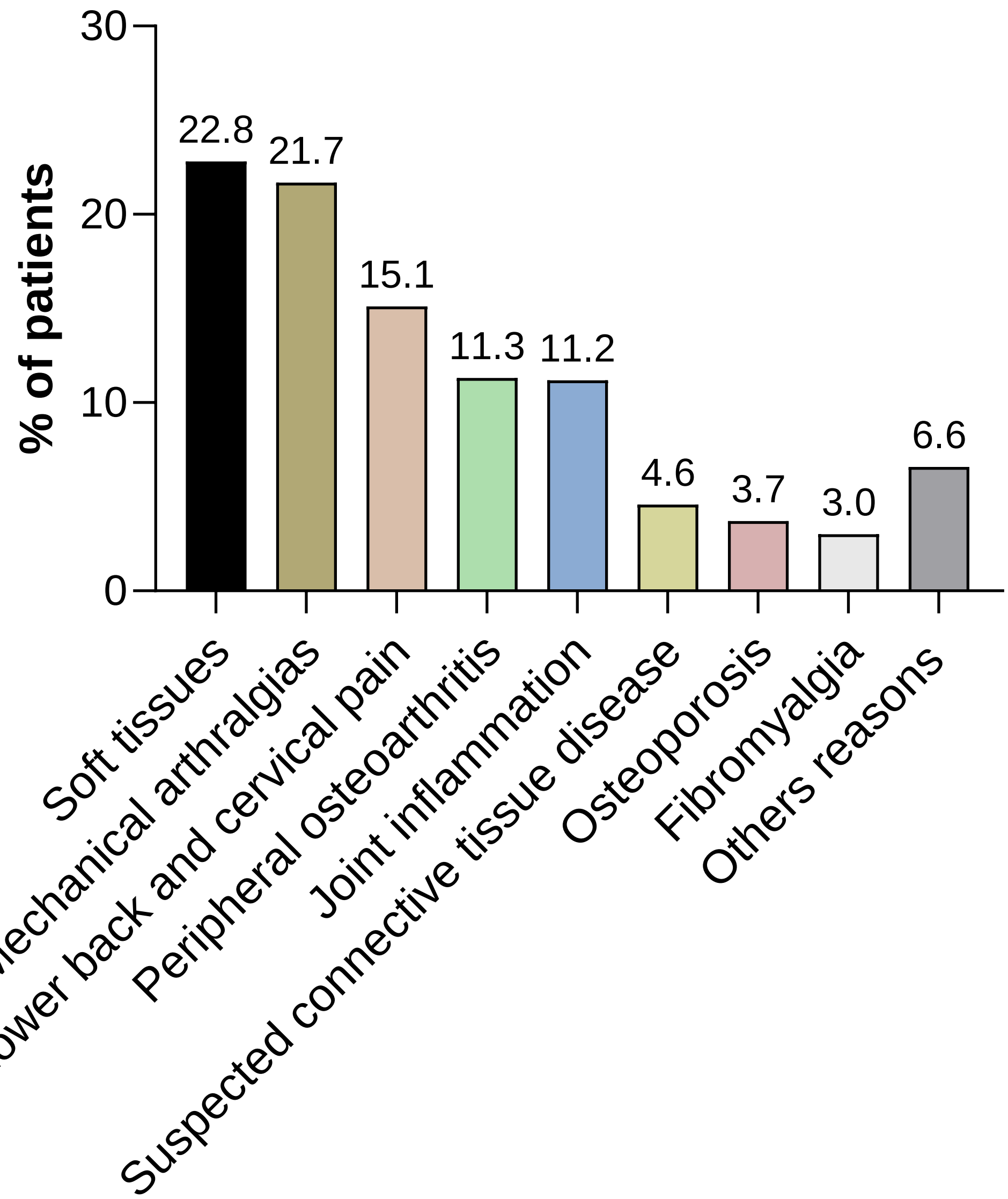

Supplementary Figure 3. Bar graph showing the distribution of suspected diagnoses among patients referred to the VIC program. Numbers above the bars indicate percentages.

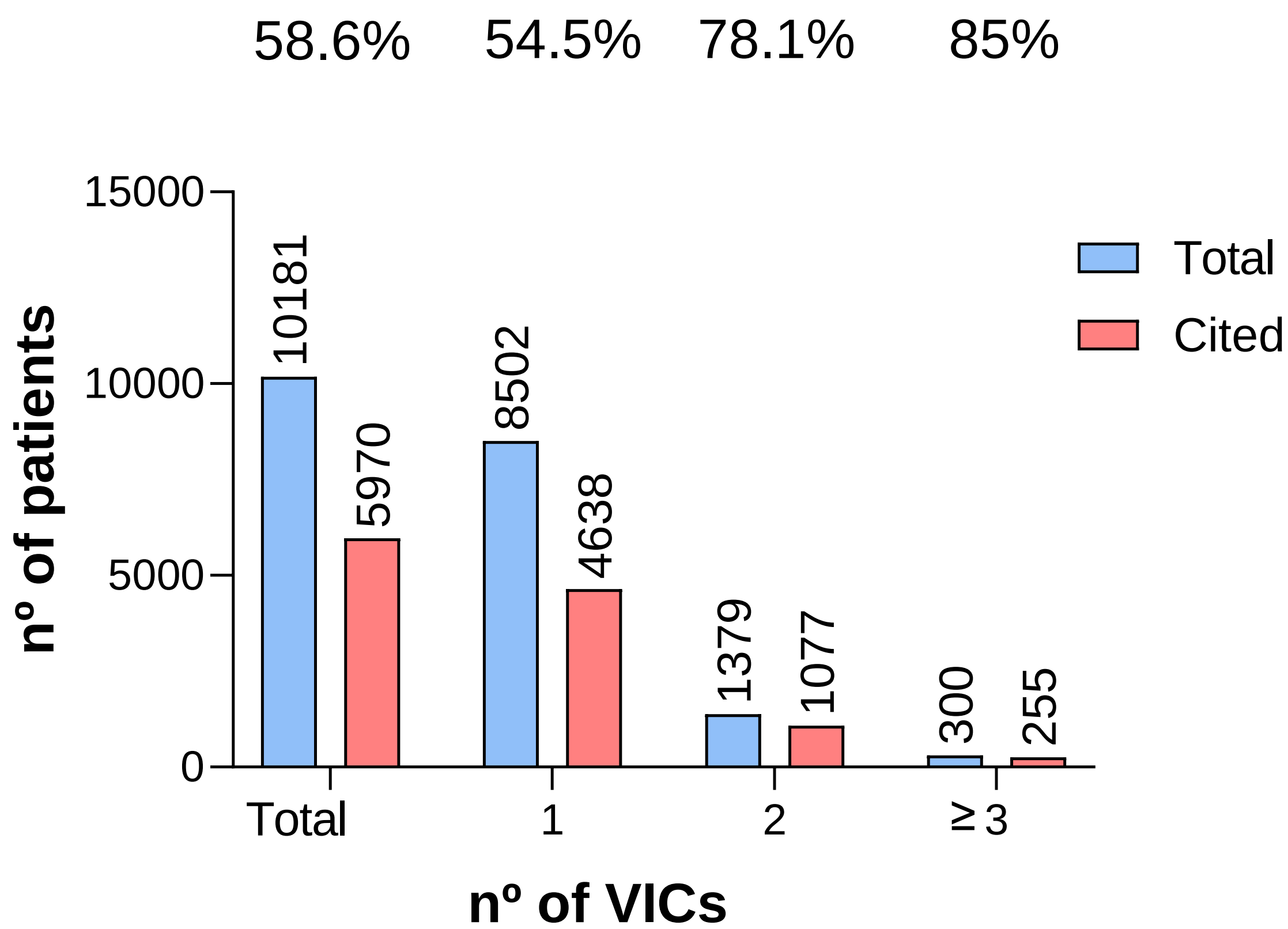

Supplementary Figure 4. Distribution of patients cited and not cited by number of VICs performed. Bar graph comparing total patients and cited patients across categories of VICs (1, 2, ≥3). Numbers above each bar show patient counts; percentages above category groups indicate the proportion of cited patients within each category.

Supplementary Text:

The analysis of economic impact of the VIC program incorporated the following parameters: the average employer cost of a specialist in the Canary Islands Health Service (CHS) [18]; the average hourly employer cost of a worker in the Canary Islands [21]; the average cost of public transport (bus) from the health area to the hospital [19]; the proportion of patients of working age (25–65 years) referred through VIC; and the unemployment rate in the Canary Islands among the active population in 2021 [20]. For in-person consultations, an average waiting time of 30 minutes in the waiting room and 30 minutes for the consultation itself was assumed.

Per-consultation personnel costs were estimated by converting the specialist's employer cost to an hourly rate and multiplying by the consultation duration (30 minutes). Patient time costs were calculated by multiplying the average employer cost per hour for Canary Islands workers by the total patient time lost per avoided in-person visit (2.5 hours, comprising average travel time, 30-minute waiting room time, and 30-minute consultation). This gross productivity loss was then adjusted for employment status by applying the 2021 unemployment rate, so that only economically active patients contributed to lost-work estimates.

Travel costs were incorporated using the average public transport fare for the route from the health area to the hospital as the per-visit travel expense. Costs associated with private car travel, parking, or ancillary expenses were not included.

The per-visit economic cost was calculated as the sum of specialist time cost, unemployment-adjusted patient productivity loss, and public-transport travel cost. Programme-level outcomes were obtained by aggregating per-visit costs across the number of visits or patients included in the VIC program, as reported in Table 4. Primary estimates refer to first consultations using the 30-minute consultation and 30-minute waiting time assumptions.

Key assumptions and limitations are reported alongside the estimates. Working age was defined as 25–65 years, and productivity losses were limited to this subgroup. The 2021 unemployment rate was applied uniformly across the working-age cohort; heterogeneous employment patterns within subgroups were not modelled. Travel costs were restricted to public-transport bus fares, potentially underestimating the burden for patients using private vehicles. Deterministic sensitivity analyses were performed by varying principal parameters (specialist hourly cost, worker hourly cost, travel cost, waiting and consultation times, and unemployment rate) across plausible ranges to assess robustness. Results of these checks and the final aggregated figures are presented in Table 4.
